# Single-Dose Intranasal or Intramuscular Administration of Simian Adenovirus-Based H1N1 Vaccine Induces a Robust Humoral Response and Complete Protection in Mice

**DOI:** 10.3390/v17081085

**Published:** 2025-08-05

**Authors:** Daria V. Voronina, Irina V. Vavilova, Olga V. Zubkova, Tatiana A. Ozharovskaia, Olga Popova, Anastasia S. Chugunova, Polina P. Goldovskaya, Denis I. Zrelkin, Daria M. Savina, Irina A. Favorskaya, Dmitry V. Shcheblyakov, Denis Y. Logunov, Alexandr L. Gintsburg

**Affiliations:** N. F. Gamaleya Federal Research Center for Epidemiology & Microbiology, Ministry of Health, Moscow 123098, Russia; vavilovairinav@yandex.ru (I.V.V.); olga-zubkova@yandex.ru (O.V.Z.); t.ozh@yandex.ru (T.A.O.); olga.popova31@yandex.ru (O.P.); a.s.chugunova@mail.ru (A.S.C.); goldovskaya00@mail.ru (P.P.G.); aleza4striker@gmail.com (D.I.Z.); daria.m.savina@gmail.com (D.M.S.); irina.favorskaya@gmail.com (I.A.F.); sdmitryv@yahoo.com (D.V.S.); ldenisy@gmail.com (D.Y.L.); gintsburg@gamaleya.org (A.L.G.)

**Keywords:** SAd25, ChAd68, adenoviral vector, influenza, H1N1, humoral immunity

## Abstract

Despite the widespread accessibility of vaccines and antivirals, seasonal influenza virus epidemics continue to pose a threat to public health. In this study, we constructed a recombinant replication-deficient simian adenovirus type 25 vector carrying the full-length hemagglutinin (HA) of the H1N1 influenza virus, named rSAd25-H1. Both systemic and mucosal humoral immune responses, as well as the protective efficacy, were assessed in mice immunized via the intramuscular (IM) or intranasal (IN) route. A single-dose IM or IN administration of rSAd25-H1 elicited a robust systemic IgG antibody response, including hemagglutination inhibition antibodies. As expected, only IN immunization was able to induce IgA production in serum and respiratory mucosa. Notably, a single dose of rSAd25-H1 at the highest dose (10^10^ viral particles) conferred complete protection against lethal homologous H1N1 challenge in mice despite the route of administration. These findings demonstrate the potential of simian adenovirus type 25-based vectors as a promising candidate for intranasal vaccine development targeting respiratory pathogens.

## 1. Introduction

Seasonal influenza remains a significant global public health concern, causing 3–5 million severe cases and approximately 650,000 deaths annually due to respiratory complications [1]. These seasonal epidemics are caused by influenza A virus (IAV; subtypes H1N1 and H3N2) and influenza B virus. Influenza B viruses are further classified into two lineages: Victoria and Yamagata. However, in recent years, only the Victoria lineage has been circulating in the human population [2].

The primary strategy for preventing influenza virus infection is vaccination. However, vaccine effectiveness ranges from 10% to 60%, depending on geographic region, age group, and antigenic similarity between the vaccine strains and circulating viruses [3]. Most commercially available vaccines are produced using egg-based manufacturing processes, during which the viral antigens may acquire adaptive mutations. These changes can reduce the antigenic match with circulating strains and subsequently decrease vaccine effectiveness [4]. Current influenza vaccines—whether whole inactivated, split, or subunit formulations—primarily induce a humoral immune response targeting hemagglutinin (HA), the most abundant glycoprotein on the viral surface. However, glycoproteins such as HA are challenging to produce in eggs or cell lines due to differences in glycosylation patterns, which may negatively affect the antigenicity and immunogenicity of current influenza vaccines [5]. Moreover, due to the rapid evolution of the major surface antigen HA, influenza vaccine formulations must be updated annually to maintain efficacy. The entire vaccine production process takes six months, and this timeframe can be critical during both seasonal outbreaks and potential pandemics [6]. Therefore, new and accelerated vaccine development approaches are needed to prepare for a potential influenza pandemic, enabling native HA expression in its natural conformation and rapid adaptation to emerging influenza strains.

Among the available vaccine platforms, adenoviral vectors (Ad) have been extensively studied and have demonstrated efficacy against infectious diseases in multiple clinical trials [7,8,9,10]. Ad-based vectors have a high transgene capacity, exhibit efficient transduction, and can be produced at high titers. These vectors are capable of inducing both innate and adaptive T and B cell immune responses [11,12,13]. Overall, Ad-based platforms hold significant potential for vaccine development, including for respiratory viral infections, due to their strong ability to transduce airway epithelial cells [14].

Currently approved influenza vaccines are typically administered via the intramuscular route, which predominantly induces a systemic immune response. However, in the case of respiratory pathogens, intranasal immunization offers distinct advantages, as it leads to the induction of both local (mucosal) and systemic immunity [15]. Furthermore, intranasal immunization provides several social and economic advantages. The intranasal route is non-invasive, making it more acceptable to patients, and does not require highly trained personnel for administration—an important consideration for low-resource settings in developing countries. These benefits make intranasal vaccination an attractive alternative to conventional intramuscular delivery.

In this study, we engineered a replication-deficient simian adenovirus type 25 (SAd25) vector expressing the full-length HA protein of the H1N1 influenza virus, designated rSAd25-H1. We evaluated the systemic humoral response, local mucosal immunity, and in vivo protection elicited by a single-shot immunization with different doses of rSAd25-H1. A single intranasal administration of 10^10^ viral particles (vp) of rSAd25-H1 induced a robust systemic and mucosal humoral immune response in mice and provided 100% protection against morbidity and mortality following challenge with a high lethal dose of H1N1. Our study highlights the potential of the SAd25-based platform as a promising candidate for the development of intranasal vaccines against respiratory viral infections.

## 2. Materials and Methods

### 2.1. Cells and Viruses

HEK293 (human embryonic kidney cells, expressing genes of the E1 region of human Ad type 5) and A549 (adenocarcinomic human alveolar basal epithelial cells) cell lines were obtained from the Russian Collection of Vertebrate Cell Lines, Russia. The MDCK (Madin–Darby canine kidney) cell line was kindly gifted by Dr. of Medical Science Elena I. Burtseva (D.I. Ivanovsky Research Institute of Virology, Moscow, Russian Federation). The cell lines were cultured in Dulbecco’s Modified Eagle’s Medium (DMEM) (Hyclone, Logan, UT, USA) containing 10% fetal bovine serum (BioSera, Cholet, France), penicillin (100 U/mL), and streptomycin (100 ug/mL) under standard conditions (37 °C, 5% CO_2_, humidified atmosphere).

A/Victoria/2570/2019 (H1N1) was used for IAV challenge in mice and was propagated in MDCK cells using standard techniques.

### 2.2. Generation of Replication-Defective Recombinant SAd25 Vector (rSAd25-H1)

The full-length HA protein (including transmembrane and cytoplasmic domains) derived from A/Wisconsin/588/2019 (H1N1) was used as the model antigen for the generation of rSAd25-H1. The nucleotide sequence of the HA gene was acquired from the GISAID database (accession number EPI1661231). It was codon-optimized for mammalian expression and synthesized by Evrogen, Moscow, Russia.

Production of the recombinant SAd25 was performed as described previously [16,17]. Briefly, pSAd25-H1, a plasmid containing the SAd25 genome with deletions of E1 and E3 regions and with the HA gene inserted into the E1 region, was constructed using standard molecular cloning techniques. Then, HEK293 cells were transfected with linearized pSAd25-H1, and the development of a cytopathic effect was monitored. Purification of the obtained rSAd25-H1 was carried out by double ultracentrifugation in a CsCl gradient.

### 2.3. Western Blotting

A549 cells were seeded into 6-well culture plates (1.5 × 10^6^ cells per well) and incubated overnight under standard conditions. The following day, the cells were transduced with 1.5 × 10^9^ vp of rSAd25-H1 and incubated at 37 °C, in a humidified atmosphere containing 5% CO_2_ for 72 h. Then, the cells were collected and lysed by the addition of Luciferase Cell Culture Lysis 5X Reagent (Promega, Madison, WI, USA).

Protein samples were separated under reducing and denaturing conditions using Any kD™ Mini-PROTEAN^®^ TGX™ Precast Gel (Bio-Rad, Hercules, CA, USA) and transferred to a 0.2 µm pore size nitrocellulose membrane (Bio-Rad, Hercules, CA, USA). After membrane blocking with 5% skimmed milk in PBS containing 0.05% Tween-20 (TPBS), primary polyclonal anti-H1 antibodies (Cat: 11055-T62, Sino Biological, Beijing, China) were added. Next, the membrane was incubated with horseradish peroxidase (HRP)-conjugated anti-rabbit IgG secondary antibodies (Cat: SSA003, Sino Biological, Beijing, China). Detection was carried out using Clarity™ Western ECL substrate (Bio-Rad, Hercules, CA, USA).

### 2.4. Immunization and Blood Serum Collection

**Figure 1:**
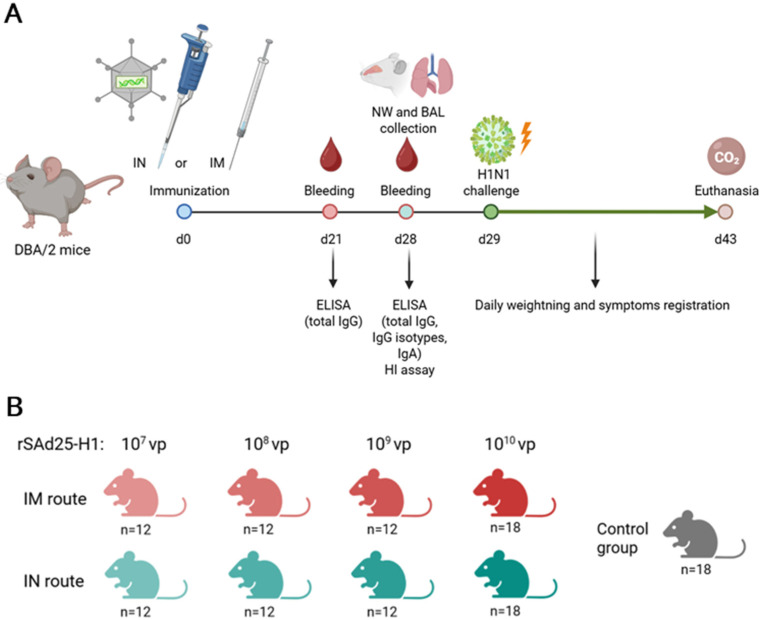
(**A**) Schematic representation of the experimental design. (**B**) Overview of experimental groups, including route of administration and rSAd25-H1 dose, with the total number of mice per group. Separate groups of mice (*n* = 6 per group) were used for blood serum collection, NW and BAL collection, and IAV challenge.

Specific pathogen-free female DBA/2 mice were obtained from an onsite animal breeding facility and housed under specific pathogen-free conditions with free access to food and water. The mice (*n* = 6 per group) received a single immunization with rSAd25-H1 via either the intramuscular (IM) or intranasal (IN) route at doses of 10^10^, 10^9^, 10^8^, or 10^7^ vp. IM immunization was performed by injecting 100 µL of the vaccine solution into the thigh muscles of the hind limbs (50 µL per limb). IN immunization was carried out by administering a total volume of 50 µL (25 µL per nostril). The mice were anesthetized with inhaled isoflurane prior to IN administration. The control group received phosphate-buffered saline (PBS) via the IM route. All experimental procedures were conducted in accordance with the Directive 2010/63/EU and FELASA recommendations and were approved by the local animal ethics committee (protocol #30, 28 October 2022). The experimental design and vaccination groups are presented in Figure 1.

Blood samples were collected on days 21 and 28 post-immunization to assess serum IgG and IgA levels. Serum samples were obtained from six animals and stored at −20 °C until further analysis.

### 2.5. Nasal Wash (NW) and Bronchoalveolar Lavage (BAL) Collection

DBA/2 mice (*n* = 6 per group) were immunized with 10^10^ vp of rSAd25-H1 via the IM or IN route; the control group received PBS intramuscularly (Figure 1). NW and BAL samples were collected on day 28 post-immunization, as previously described [18]. The samples were obtained and stored at −20 °C until further analysis.

### 2.6. Enzyme-Linked Immunosorbent Assay (ELISA)

Polystyrene microplates (Corning, New York, NY, USA) were coated overnight at 4 °C with 1 µg/mL of Influenza A (H1N1) pdm 09 control antigen (A/Victoria/2570/2019; FR-1820, International Reagent Resource, Manassas, VA, USA) in carbonate–bicarbonate buffer (pH 9.6). The plates were then washed three times with TPBS and blocked for 1 h at 37 °C with blocking buffer (TPBS supplemented with 1% *w*/*v* casein). After blocking, the plates were rinsed three times with TPBS, and serial dilutions of serum, NW, or BAL samples prepared in blocking buffer were added to the wells and incubated for 1 h at 37 °C.

After four washes with TPBS, bound antibodies were detected using secondary HRP-conjugated anti-mouse antibodies specific for total IgG, IgA, and IgG subtypes (IgG1, IgG2a, IgG2b, and IgG3), all used at a 1:5000 dilution (Abcam, Cambridge, UK). Following five additional washes, 3,3′,5,5′-tetramethylbenzidine (Bio-Rad, Hercules, CA, USA) was added as a substrate. After 15 min, the reaction was stopped by the addition of 1 M H_2_SO_4_, and the absorbance was measured at 450 nm (OD_450_). The Ab titer was determined as the highest dilution of the serum at which the OD_450_ of the test sample exceeded that of the control serum two-fold or more at the same dilution. All samples were analyzed in duplicates, and the mean values were calculated.

### 2.7. Hemagglutination Inhibition (HI) Assay

The HI assay was performed as described previously [19]. Briefly, influenza A (H1N1) pdm09 control antigen (A/Victoria/2570/2019) (FR-1820, International Reagent Resource, Manassas, VA, USA) was used as the source of specific antigen. Mouse sera were treated with a receptor-destroying enzyme (RDE II, Denka-Seiken, Tokyo, Japan) for 18 h and heat-inactivated. Serial two-fold dilutions of the serum samples were mixed with an equal volume of the control antigen (4 hemagglutinating units per 25 µL) in a 96-well U-bottom plate (Medpolymer, Saint Petersburg, Russia) and incubated at room temperature for 1 h; then, 50 µL of 0.75% human type O erythrocyte solution was added to each well, followed by incubation at room temperature for 1 more hour. The HI titer was determined as the highest serum dilution that caused complete inhibition of the control antigen.

### 2.8. Influenza Virus Challenge

The mice (*n* = 6 per group) were challenged intranasally with 1300 LD_50_ of mouse-adapted A/Victoria/2570/2019 (H1N1) in a total volume of 50 µL (25 µL per nostril). The animals were anesthetized with inhaled isoflurane prior to IAV challenge. The mice were monitored daily for clinical signs and body weight loss. Clinical symptoms were assessed according to the following scoring scale: score 0—no symptoms; score 1—slightly ruffled fur; score 2—ruffled fur and decreased activity OR 10–15% weight loss; score 3—ruffled fur, decreased activity, and rapid breathing OR 15–20% weight loss; score 4—ruffled fur, minimal activity, hunched posture, rapid and/or labored breathing OR 20–25% weight loss; and score 5—more than 25% weight loss—euthanasia endpoint.

### 2.9. Statistical Analysis

Statistical analysis was performed using GraphPad Prism 8.0.1 software. Antibody titer data are presented as geometric mean ± 95% CI. Statistical differences between groups were analyzed using the Kruskal–Wallis test followed by Dunn’s multiple comparisons test (****, *p* < 0.0001; ***, *p* < 0.001; **, *p* < 0.01; *, *p* < 0.05; ns, *p* ≥ 0.05).

## 3. Results

### 3.1. Generation of a Simian Adenovirus Type 25 Vector Expressing the HA Gene of H1N1 IAV

**Figure 2:**
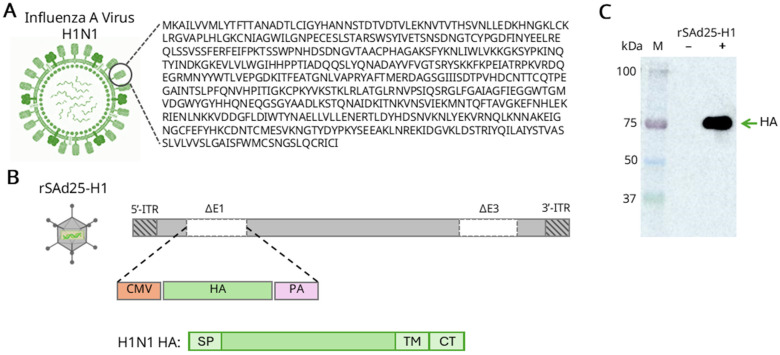
Generation and characterization of rSAd25-H1—recombinant replication-defective simian adenovirus type 25 expressing the HA gene of IAV A/Wisconsin/588/2019 (H1N1). (**A**) Schematic representation of the H1N1 IAV virion structure and the corresponding HA amino acid sequence. (**B**) A schematic diagram of the rSAd25-H1 recombinant genome generated by homologous recombination: ITR, inverted terminal repeat; CMV, cytomegalovirus promoter; PA, simian virus 40 polyadenylation signal; SP, HA signal peptide; TM, transmembrane domain; CT, cytoplasmic tail. (**C**) Western blot analysis of HA protein expression in lysates of A549 cells transduced with rSAd25-H1 or mock-infected. The membrane was probed with anti-HA antibodies. The band with an approximate molecular weight of 75 kDa corresponds to a monomeric glycosylated form of the HA protein.

The codon-optimized nucleotide sequence encoding the full-length HA protein of A/Wisconsin/588/2019 (H1N1) IAV (Figure 2A), under the control of a cytomegalovirus (CMV) promoter and a polyadenylation signal (PA), was inserted into the E1 region of the adenoviral genome via homologous recombination. This resulted in the generation of a recombinant replication-deficient simian adenovirus type 25 vector, expressing HA, designated rSAd25-H1 (Figure 2B). Expression of HA, which has a predicted molecular weight of 63 kDa, was confirmed by Western blot analysis of lysates from rSAd25-H1-transduced A549 cells using polyclonal anti-HA antibodies (Abs) (Figure 2C). A single band with an approximate molecular weight of 75 kDa was detected, indicating glycosylation of the HA protein expressed in mammalian cells.

### 3.2. rSAd25-H1 Elicits a Systemic Humoral Immune Response Following a Single Immunization

To assess the immunogenicity of rSAd25-H1, DBA/2 mice received a single IM or IN administration of varying doses of rSAd25-H1, as shown in Figure 1B. Systemic IgG antibody responses, as well as hemagglutination inhibition (HI) antibody levels, were evaluated.

**Figure 3:**
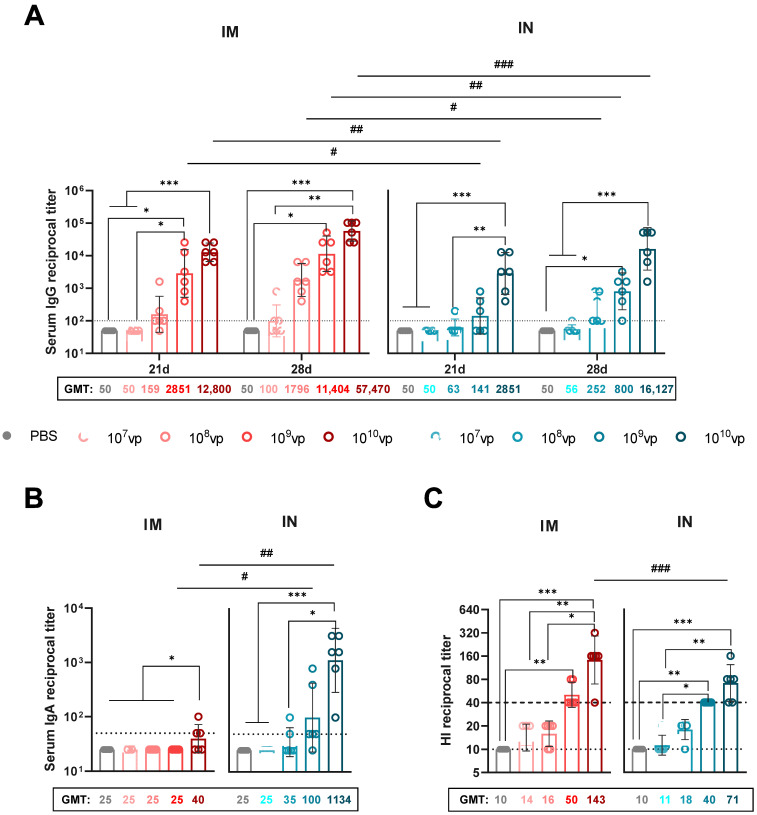
Characterization of systemic humoral immune responses elicited by a single immunization with rSAd25-H1. DBA/2 mice were immunized intramuscularly (IM) or intranasally (IN) with 10^7^–10^10^ vp of rSAd25-H1. Control mice received an equal volume of PBS via the IM route. (**A**) HA-specific serum IgG endpoint titers measured by ELISA on days 21 and 28 following IM or IN immunization. (**B**) Serum HA-specific IgA endpoint titers on day 28 post-vaccination. (**C**) Hemagglutination inhibition (HI) antibody titers against H1N1 IAV in serum samples collected on day 28 post-vaccination. The dashed line indicates an HI titer ≥ 1:40, considered a correlate of protection in humans. Data are presented as the geometric mean with 95% confidence intervals (CIs). The dotted lines indicate the input serum dilution and represent the lower limit of detection for each assay. Significant differences between different doses within the IM or IN group are indicated with asterisks: ***, *p* <0.001; **, *p* < 0.01; *, *p* < 0.05 (the analysis was performed using one-way ANOVA with Dunn’s multiple comparison test). Statistically significant intergroup differences are indicated with hashes: ###, *p* <0.001; ##, *p* < 0.01; #, *p* < 0.05 (calculated using the Mann–Whitney test).

HA-specific serum IgG levels were measured by ELISA on days 21 and 28 post-immunization using the full-length H1N1 HA protein as the antigen, which corresponds to the HA sequence encoded by rSAd25-H1 (Figure 3A). Overall, the systemic IgG response increased in a dose-dependent manner and was significantly higher following IM immunization compared to IN delivery. On day 21, a statistically significant difference in serum IgG endpoint titers was observed for the 10^9^ vp and 10^10^ vp doses: the geometric mean titers (GMTs) were approximately 20-fold and 4.5-fold higher, respectively, for IM versus IN administration. The most robust and statistically significant increase in the humoral response was observed on day 28 at a dose of 10^10^ vp. At this time point, GMTs following IM administration of rSAd25-H1 were approximately 7-fold higher for 10^8^ vp, ~14-fold higher for 10^9^ vp, and ~3.5-fold higher for 10^10^ vp compared to their corresponding IN groups.

IgA antibody levels were measured by ELISA in the serum of the immunized mice on day 28 post-vaccination (Figure 3B). HA-specific serum IgA titers increased in a dose-dependent manner and were detected at high levels in the mice immunized via the IN route. Among the groups immunized via the IM route, serum IgA was detected only in the group that received 10^10^ vp of rSAd25-H1, and at significantly lower levels compared to the IN group receiving the same dose. IN immunization resulted in a statistically significant increase in serum IgA titers at doses of 10^9^ and 10^10^ vp. Notably, the GMT for the IN group at the 10^10^ vp dose was 28.6-fold higher than that of the IM group at the same dose.

Next, we assessed the ability of rSAd25-H1 to induce HI Abs (Figure 3C). The HI antibody titer is considered the primary correlate of protection for influenza vaccines. Specifically, an HI titer of ≥1:40 is generally accepted as the threshold for protective immunity against influenza virus infection [20]. Following a single IM immunization with rSAd25-H1, HI titers in the mouse serum against A/Victoria/2570/2019 (H1N1) increased in a dose-dependent manner. At doses of 10^9^ and 10^10^ vp, 100% of the mice achieved HI titers ≥ 1:40, with median titers of 1:50.4 and 1:142.5, respectively. At lower doses of rSAd25-H1 (10^7^ and 10^8^ vp), vaccination induced HI Abs with titers of ≥1:15.

Similarly, a single IN administration of rSAd25-H1 at doses of 10^9^ and 10^10^ vp elicited HI Abs in 100% of the mice, with median titers of 1:40 and 1:71.3, respectively. A statistically significant difference in HI titers between the two routes of administration was observed only at the 10^10^ vp dose of rSAd25-H1.

In summary, a single-dose immunization with rSAd25-H1 at doses of 10^9^ and 10^10^ vp, regardless of the route of administration, elicited a strong systemic IgG antibody response, including robust HI activity. However, IN immunization resulted in higher levels of serum IgA compared to the intramuscular IM route.

### 3.3. Administration of rSAd25-H1 Induces a Robust Mucosal Humoral Response

**Figure 4:**
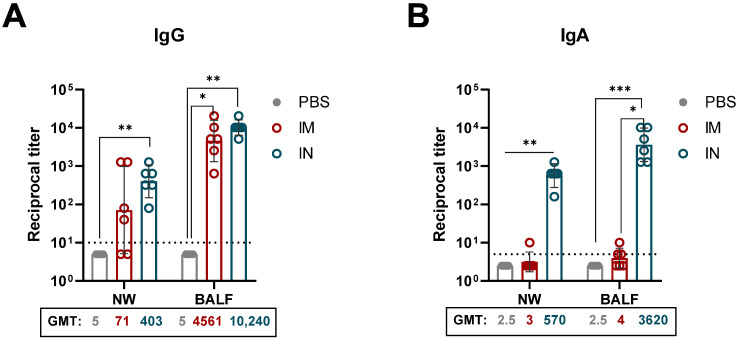
Respiratory mucosal humoral immune responses elicited by a single immunization with rSAd25-H1. HA-specific mucosal IgG (**A**) and IgA (**B**) titers were determined by ELISA in NWs and BALs collected from DBA/2 mice on day 28 post-vaccination with 10^10^ vp of rSAd25-H1 administered via the IM or IN route. Data are presented as geometric means with 95% CIs. The dotted lines indicate the input serum dilution and represent the lower limit of detection for each assay. Statistical analysis was performed using one-way ANOVA with Dunn’s multiple comparison test: ***, *p* < 0.001; **, *p* < 0.01; *, *p* < 0.05.

The respiratory mucosal immune system plays a critical role in preventing viral entry and infection within the respiratory tract. To evaluate the induction of mucosal immunity, NWs and BAL fluids were collected on day 28 following a single IM or IN immunization with 10^10^ vp of rSAd25-H1 (Figure 4). The levels of H1 HA-specific IgG and IgA Abs were evaluated. Both the IN and IM administration routes induced IgG responses in the mucosa, with no statistically significant difference observed between the groups. As expected, high titers of HA-specific IgA were detected in the IN-immunized mice (GMT 570.2 in NWs and GMT 3620.4 in BALs), and no detectable levels of HA-specific secretory IgA were found in the mucosal samples from the IM-immunized mice.

Taken together, these findings indicate that a single IN immunization with rSAd25-H1 effectively induces strong mucosal immunity, as evidenced by increased levels of mucosal IgG and IgA antibodies.

### 3.4. Route of rSAd25-H1 Administration Affects IgG Isotype Profiles and Th1/Th2 Polarization

**Figure 5:**
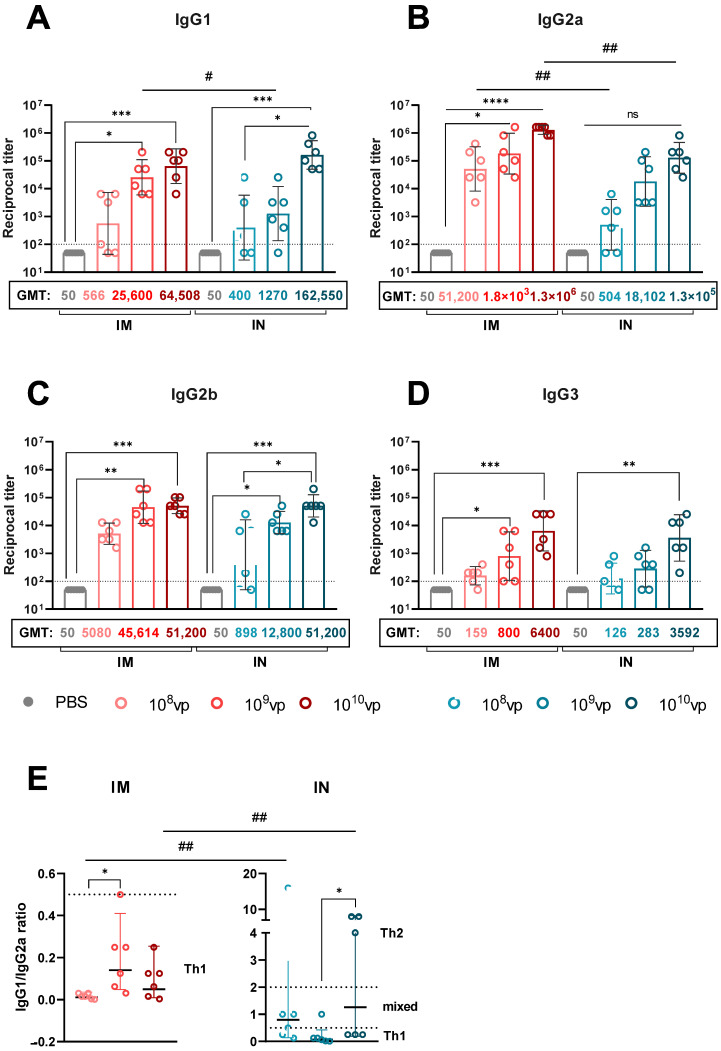
Analysis of HA-specific IgG isotypes in rSAd25-H1-immunized mice. IgG1 (**A**), IgG2a (**B**), IgG2b (**C**), and IgG3 (**D**) endpoint titers were measured by ELISA in serum samples collected on day 28 post-vaccination from mice immunized IM or IN with rSAd25-H1 at doses of 10^8^–10^10^ vp. Dotted lines indicate input serum dilutions and represent the lower limit of detection for each assay. (**E**) Ratios of IgG1/IgG2a in serum following IM or IN immunization with rSAd25-H1. Dotted lines at 0.5 and 2.0 indicate three different immune response profiles: <0.5—Th1-biased; 0.5–2.0—Th1/Th2-balanced; >2.0—Th2-polarized. Data are presented as geometric means with 95% CIs. Significant differences between different doses within the IM or IN group are indicated with asterisks: (****, *p* < 0.0001; ***, *p* <0.001; **, *p* < 0.01; *, *p* < 0.05 (the analysis was performed using one-way ANOVA with Dunn’s multiple comparison test). Statistically significant intergroup differences are indicated with hashes: ##, *p* < 0.01; #, *p* < 0.05 (calculated using the Mann–Whitney test).

To further characterize the humoral immune response, we analyzed the H1N1 HA-specific IgG isotype profiles induced by rSAd25-H1 administered IM or IN at varying doses (Figure 5). It is well established that different IgG isotypes exhibit varying affinities for Fc-gamma receptors (FcγRs), which can influence the efficacy of vaccines in mediating protection against pathogens [21]. Moreover, isotype profiles have been shown to correlate with Th1 and Th2 cytokine profiles. In mice, IgG1 is generally associated with Th2 (humoral) responses, while IgG2a is indicative of Th1 (cellular) responses [22]. A ratio of IgG1/IgG2a < 0.5 suggests a Th1-biased response due to elevated IgG2a levels. A ratio between 0.5 and 2.0 indicates a mixed or balanced Th1/Th2 response, whereas an IgG1/IgG2a ratio > 2.0 reflects Th2 polarization, characterized by increased IgG1 production [23,24,25].

Immunization with rSAd25-H1 via both routes induced high levels of IgG1, IgG2a, and IgG2b Abs, and significantly lower levels of IgG3, in a dose-dependent manner (Figure 5A–D). IM administration resulted in a consistent IgG1/IgG2a ratio of <0.5 across all the dose groups, indicating a Th1-biased response (Figure 5E). In contrast, the IgG1/IgG2a ratio in the IN-immunized mice varied depending on the dose (Figure 5E): 10^8^ and 10^10^ vp induced balanced Th1/Th2 responses, while 10^9^ vp resulted in a Th1-biased profile. Notably, the observed differences in Th1/Th2 polarization between the IM and IN groups were primarily driven by higher IgG2a levels following IM immunization, rather than elevated IgG1 titers after IN vaccination. Taken together, these data indicate that IM delivery of rSAd25-H1 promotes Th1-biased immunity, whereas IN administration primarily elicits a more balanced Th1/Th2 response.

### 3.5. A Single Immunization with rSAd25-H1 Protects Mice from Lethal H1N1 IAV Infection

**Figure 6:**
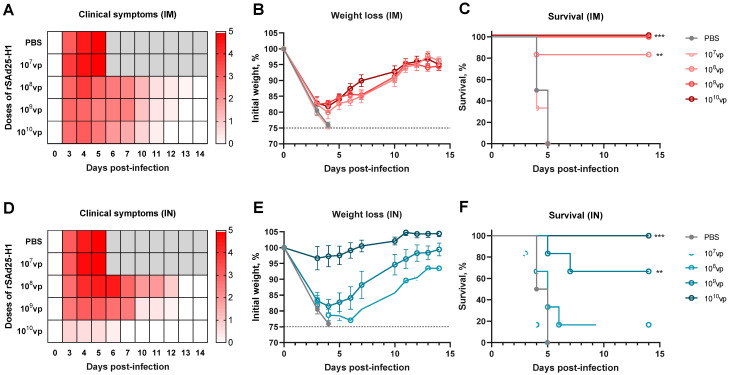
In vivo protective efficacy of rSAd25-H1 delivered either IM or IN. DBA/2 mice (*n* = 6 per group) were immunized IM or IN with rSAd25-H1 at doses ranging from 10^7^ to 10^10^ vp. On day 29 post-vaccination, the animals were challenged with 1300 LD_50_ of homologous H1N1 IAV. Clinical signs (**A**,**D**), body weight changes (**B**,**E**), and survival rates (**C**,**F**) were monitored for 14 days post-infection. Body weight curves are presented as mean value ± standard error of the mean (SEM). The significance of survival differences was calculated using the log-rank (Mantel–Cox) test: **, *p* < 0.01; ***, *p* < 0.001.

To evaluate the protective efficacy of a single-shot rSAd25-H1 vaccination against lethal viral challenge, DBA/2 mice were immunized either IM or IN with rSAd25-H1 at doses ranging from 10^7^ to 10^10^ vp as described above (Figure 1). On day 29 post-vaccination, the mice (*n* = 6 per group) were challenged with 1300 LD_50_ of mouse-adapted H1N1 IAV (A/Victoria/2570/2019), which expresses the same HA variant as that encoded by rSAd25-H1. The animals were monitored daily for 14 days post-infection for signs of morbidity (body weight loss and clinical symptoms) and mortality (survival rate), after which the surviving animals were euthanized. The mice that experienced a loss of more than 25% of their initial body weight were humanely euthanized in accordance with ethical guidelines (Figure 6).

Challenge with homologous H1N1 resulted in symptomatic infection in the control group and all the IM-immunized groups (Figure 6A). The IM-vaccinated animals exhibited significant weight loss, comparable to that of the control mice, reaching up to 25% of their initial body weight (Figure 6B). Despite this substantial weight loss, IM immunization with 10^9^ and 10^10^ vp of rSAd25-H1 conferred 100% protection from mortality, while 10^8^ vp provided approximately 83% survival (Figure 6C). In contrast, the unvaccinated animals and those that received 10^7^ vp of rSAd25-H1 succumbed to infection and were either humanely euthanized or died by day 6 post-infection.

The IN-immunized mice demonstrated a more pronounced dose-dependent pattern in terms of clinical symptoms and weight loss (Figure 6D,E). All the mice that received 10^10^ vp of rSAd25-H1 via the IN route survived the lethal H1N1 challenge and exhibited minimal or no weight loss (Figure 6E,F). The mice vaccinated IN with 10^9^ vp experienced up to 20% weight loss but began to recover after day 4 post-challenge, with a survival rate of approximately 67%. Doses of 10^8^ vp or lower administered via the IN route did not protect the animals against the lethal challenge with a relatively high dose (1300 LD_50_) of H1N1. Taken together, these results indicate that rSAd25-H1, regardless of the route of administration, provides dose-dependent protection against mortality caused by high-dose H1N1 IAV challenge.

## 4. Discussion

The recent COVID-19 pandemic has underscored the urgent need for improved preparedness against future pandemics. Among the pathogens with pandemic potential, influenza A virus stands out as a particularly significant threat. Since the 20th century, IAV has been responsible for several pandemics, with the most devastating being the 1918 “Spanish flu”, which claimed an estimated 40 to 100 million lives [26]. To effectively control infection, it is essential to adopt preventive measures through the use of targeted vaccines. Effective control of influenza requires the implementation of preventive strategies, with vaccination remaining the most efficient tool. Therefore, ongoing development of innovative immunization platforms and novel vaccine strategies is essential.

Licensed influenza vaccines, such as whole inactivated or split/subunit vaccines, primarily induce humoral immune responses. In contrast, Ad-based vaccines are known to elicit both antibody and T-cell responses [27]. Furthermore, replication-deficient Ad vectors are safe and well-characterized, and their advantages include a significant decrease in the possibility of mutations in the vaccine antigen during the manufacturing process [28].

During the COVID-19 pandemic, both human and chimpanzee Ad-vectored vaccine platforms were actively developed and deployed, raising concerns about pre-existing immunity to Ad vectors, which may reduce vaccine efficacy [29]. The negative impact of pre-existing vector immunity was demonstrated using human Ad type 5 (Ad5): preexposure to Ad5 abolished the transgene-specific antibody response when delivered via the intramuscular route in both mice and non-human primates [30,31]. Various strategies have been employed to overcome pre-existing immunity to Ad vectors, one of which is the use of non-human Ads. In the present work, we developed a novel influenza vaccine candidate based on simian adenovirus type 25 (SAd25), also known as ChAd68, which encodes the HA protein of A (H1N1) IAV—rSAd25-H1. SAd25 has low seroprevalence in humans and minimal cross-reactivity with neutralizing antibodies specific to human Ads, thereby circumventing vector-specific immunity [32,33]. The SAd25 vector system has been investigated as a promising tool for combating various infectious diseases, including respiratory viruses such as SARS-CoV-2, influenza, and respiratory syncytial virus [34,35,36,37,38]. Furthermore, it has been studied for its potential application in the treatment of solid tumors [39].

Most currently approved influenza vaccines are administered intramuscularly and fail to stimulate robust T-cell or mucosal immunity—both critical for protection against respiratory viruses [40,41]. Alternative approaches, such as using viral vectors or adjuvants and modifying the route of administration, may help skew responses toward mucosal IgA production or cellular immunity. Many studies have demonstrated the immunogenic potential and efficacy of intranasally administered anti-influenza vaccines, including those based on Ad vectors [42,43,44,45,46,47,48,49,50,51,52,53,54]. Moreover, intranasal delivery of Ad-based vaccines may overcome pre-existing immunity to the vector [30,34]. Therefore, in this study, we evaluated serum and mucosal antibody responses, as well as in vivo protective efficacy, following IM or IN administration of rSAd25-H1 in mice.

Replication-deficient Ad-vectored vaccines induce potent T- and B-cell responses to expressed antigens. In order to explore HA-specific humoral immunogenicity induced by rSAd25-H1, mice were immunized with a single dose of varying amounts of rSAd25-H1. Serum IgG endpoint titers analysis demonstrated a dose-dependent immune response regardless of the route of administration of rSAd25-H1. We showed that IM immunization with rSAd25-H1 induces significantly higher levels of systemic IgG compared to IN delivery. This finding is consistent with a previous study on the use of chimpanzee-derived Ad-based vaccine for the prevention of IAV [54]. A similar tendency was observed for HI Abs titers, a key correlate of protection for influenza vaccines: IM administration of 10^10^ vp of rSAd25-H1 induced significantly higher HI Abs titers. Our study showed that IN vaccination at the same doses as IM immunization (10^9^ and 10^10^ vp) induced sufficient levels of HI Abs titers (≥1:40).

We also characterized the IgG isotype profile, given that serum IgG isotypes differ in their mechanisms of virus elimination. They not only rely on direct neutralization of pathogens but also on complement activation and Fc-effector functions [21]. Murine IgG2a antibodies are more effective at recruiting effector cells and activating the complement system efficiently [55,56]. IgG2a and IgG2b bind to all activating Fc receptors, with IgG2a having the highest affinity for most of them. In contrast, IgG1 interacts with only one activating receptor, FcγRIII [57]. Additionally, IgG2a is thought to be the predominant IgG isotype that confers protection against viral infections [58,59]. We demonstrated that rSAd25-H1 is able to induce high levels of IgG1, IgG2a, and IgG2b isotypes regardless of the administration route. However, a significant difference was observed between the two delivery routes for IgG2a levels, but not for IgG1: IM immunization resulted in higher titers of IgG2a compared to IN vaccination. Previously, it was shown that the route of administration robustly affects the IgG2a response, with only minor effects on the IgG1 isotype [60].

Secretory IgA antibodies on the surface of the respiratory mucosa play an important role in preventing airborne diseases, as they act as a first line of defense at the entry point of respiratory infections [61]. We observed that IN vaccination with rSAd25-H1 led to robust IgA production both in serum and in the respiratory mucosa. These results can be explained by the fact that IgA class switching in the airways primarily occurs in nasopharynx-associated lymphoid tissues [62]. Our findings are consistent with previous studies [49,53,63,64]. Therefore, IN vaccination induces not only systemic IgG antibodies but also antigen-specific secretory IgA in the respiratory mucosa.

Moreover, IgG1 and IgG2a Abs titers have been shown to correlate with Th2 and Th1 cellular immunity, respectively [22]. Following natural infection, influenza viruses typically induce a Th1-biased adaptive immune response, characterized by the production of pro-inflammatory cytokines and the activation of macrophages, cytotoxic lymphocytes, and NK cells [65]. Vaccination with rSAd25-H1 via the IM route at any dose led to a Th1-skewed response, which is similar to what has been previously reported [23]. Intranasal immunization with rSAd25-H1, in turn, produced a balanced Th1/Th2 adaptive immune response. However, further studies are needed to fully characterize rSAd25-H1-induced T-cell immunity.

Furthermore, we demonstrated in vivo protection against challenge with matched IAV at an extremely high dose—1300 LD_50_. Notably, when administered via the IM route, rSAd25-H1 was more effective at preventing mortality, but not morbidity. Doses of 10^9^ and 10^10^ vp of rSAd25-H1 provided 100% survival of the immunized mice; however, the animals experienced weight loss and severe clinical symptoms. In contrast, IN administration of rSAd25-H1 showed complete protection against death events only in the group receiving 10^10^ vp, while the animals exhibited no significant weight loss or mild symptoms of disease. The IN vaccination with rSAd25-H1 at a dose of 10^9^ vp conferred partial protection, as four out of six mice survived the challenge. The detection of HI active Abs with a titer of ≥1:40 in the serum of the mice immunized with 10^9^ and 10^10^ vp suggests that in vivo protection may be at least partially associated with neutralizing Abs. Additionally, considering that the primary and most noticeable difference in the immune response observed between the two administration routes was in mucosal IgA levels, we speculated that the mild morbidity signs in the group of IN-immunized mice (at a dose of 10^10^ vp) compared to IM vaccination might be associated with the presence of secretory IgA at the respiratory mucosal surfaces. Moreover, the IM route, in comparison with the IN regimen, provided more pronounced protection against mortality. This might be attributed to the elevated levels of HA-specific IgG2a antibodies, which are known to effectively induce effector mechanisms, such as antibody-dependent cellular cytotoxicity, cellular phagocytosis, and complement deposition [55,56]. Nevertheless, further research is needed to elucidate the precise mechanisms of the antiviral activity of antibodies induced by rSAd25-H1.

However, our study has several limitations. We did not evaluate the long-term persistence of the antibody response. Several studies have revealed an effect of the route of administration on differences in the magnitude and kinetics of the immune response [60,66]. We did not assess the viral titer in the lungs of the infected mice, which could provide more details on the in vivo efficacy of rSAd25-H1. Furthermore, we did not thoroughly examine the T-cell-mediated adaptive immune response. A number of animal and human studies have demonstrated the role of CD4+ and CD8+ T-cell immunity in protection against influenza infection [67]. Additionally, it has been established that the CD8+ response is often cross-reactive, which is an important consideration given the high variability among influenza viruses [67]. In this regard, additional research is required to complete the immunological characterization of rSAd25-H1.

The rSAd25 vector represents a potent tool for developing future vaccines against respiratory viruses. Our findings indicate that both mucosal (intranasal) and parenteral (intramuscular) delivery of rSAd25-H1 could induce a robust systemic humoral response and provide protection against lethal influenza challenges. However, only intranasal immunization leads to the induction of respiratory mucosal immunity. In the future, mucosal immunization could be a promising strategy for both priming and boosting vaccinations against respiratory pathogens.

## Data Availability

The raw data supporting the conclusions of this article will be made available by the authors on request.
